# Advances for pharmacological activities of *Polygonum cuspidatum* - A review

**DOI:** 10.1080/13880209.2022.2158349

**Published:** 2023-01-09

**Authors:** Jia Ke, Meng-Ting Li, Shuyang Xu, Jianpeng Ma, Ming-Yuan Liu, Yan Han

**Affiliations:** a Department of Neurology, Yueyang Hospital of Integrated Traditional Chinese and Western Medicine, Shanghai University of Traditional Chinese Medicine, Shanghai, China; b Monteverde Academy Shanghai, Shanghai, China; c Multiscale Research Institute of Complex Systems, Fudan University, Shanghai, China

**Keywords:** Huzhang, anti-inflammatory, anticancer, heart protection, pharmacokinetics, Chinese herb

## Abstract

**Context:**

*Polygonum cuspidatum* Sieb. et Zucc (Polygonaceae), the root of which is included in the Chinese Pharmcopoeia under the name ‘Huzhang', has a long history as a medicinal plant and vegetable. *Polygonum cuspidatum* has been used in traditional Chinese medicine for the treatment of inflammation, hyperlipemia, etc.

**Objective:**

This article reviews the pharmacological action and the clinical applications of *Polygonum cuspidatum* and its extracts, whether *in vivo* or *in vitro*. We also summarized the main phytochemical constituents and pharmacokinetics of *Polygonum cuspidatum* and its extracts.

**Methods:**

The data were retrieved from major medical databases, such as CNKI, PubMed, and SinoMed, from 2014 to 2022. *Polygonum cuspidatum*, pharmacology, toxicity, clinical application, and pharmacokinetics were used as keywords.

**Results:**

The rhizomes, leaves, and flowers of *Polygonum cuspidatum* have different phytochemical constituents. The plant contains flavonoids, anthraquinones, and stilbenes. *Polygonum cuspidatum* and the extracts have anti-inflammatory, antioxidation, anticancer, heart protection, and other pharmacological effects. It is used in the clinics to treat dizziness, headaches, traumatic injuries, and water and fire burns.

**Conclusions:**

*Polygonum cuspidatum* has the potential to treat many diseases, such as arthritis, ulcerative colitis, asthma, and cardiac hypertrophy. It has a broad range of medicinal applications, but mainly focused on root medication; its aerial parts should receive more attention. Pharmacokinetics also need to be further investigated.

## Introduction

Traditional Chinese medicine (TCM) has been used extensively for thousands of years, and the use of herbal medicinal products has been growing rapidly in many countries (Agbabiaka et al. 2018). For example, artemisinin and its derivatives, the most effective antimalarial drugs, are extracted from the sweet wormwood plant, *Artemisia annua* Linn (Compositae) (Yang et al. 2020). Numerous researchers have studied herbal medicine. An herbal medicine may have a variety of phytochemical constituents, each of which may have a different medicinal activity, so an herb can actually have a variety of therapeutic effects at the same time. Nowadays, people use herbal medicine alone or as a supplement to treat many diseases, such as cancer (Guo et al. 2021), cardiovascular (Xu et al. 2019), cerebrovascular, and nervous system diseases (Lu et al. 2020). Moreover, as the prevalence of TCM research increases worldwide, more pharmaceutical activities will be discovered (Acquaviva et al. 2021).

*Polygonum cuspidatum* Sieb. et Zucc (Polygonaceae) is a traditional Chinese herb that grows in Asia and North America. The roots of *Polygonum cuspidatum* (PC) are listed in the Pharmacopoeia of the People’s Republic of China using the name of Huzhang. Resveratrol, polydatin, quercetin, emodin, and their derivatives are the primary active phytochemical components of PC. These phytochemical components of PC have undergone extensive research and are thought to be essential for PC’s medicinal functions (Lachowicz and Oszmiański 2019; Wang, Feng et al. 2019). Moreover, PC has been known to have anti-inflammatory (Liu et al. 2018), antioxidant (Zeng et al. 2019), antiviral (Lin et al. 2015), antimicrobial (Yang et al. 2015), neuroprotective effects (Liu et al. 2015), etc. It is seen as a potential treatment for arthritis, ulcerative colitis (Liu et al. 2018), asthma (Zeng et al. 2019), cardiac hypertrophy (Ding W et al. 2014), etc. The main objective of this review is to provide a systematic elaboration of the therapeutic effects of PC on a variety of diseases, so as to promote the understanding of PC and the development PC-derived herbal medicinal products and supplements.

## Phytochemical constituents

The phytochemical constituents isolated and identified from PC are mainly stilbenes, anthraquinones, flavonoids, and polyphenols. The distribution and contents of different types of phytochemical constituents showed remarkable differences among different plant parts of PC. Stilbene compounds, such as resveratrol and polydatin, are the main active components in PC. Anthraquinone compounds mainly include emodin and its derivatives. Stilbene and anthraquinone compounds are more concentrated in the rhizomes than in other tissues, which may explain why the PC root is used in traditional Chinese medicine. Flavonoids are mainly found in the leaves and the stems, whereas polyphenols are more concentrated in the flowers (Wang, Feng et al. 2019; Wu, Wang et al. 2019). The root of the PC is most widely used in traditional Chinese medicine to clear away heat and toxic materials. The other plant parts, such as the leaves, are also used due to the health benefits of the phytochemicals contained. The root, leaves, flowers, rhizomes, and fibers of PC can all be used as medicinal proposes.

## Pharmacological activities

### Anti-inflammation effect

**Figure 1.**
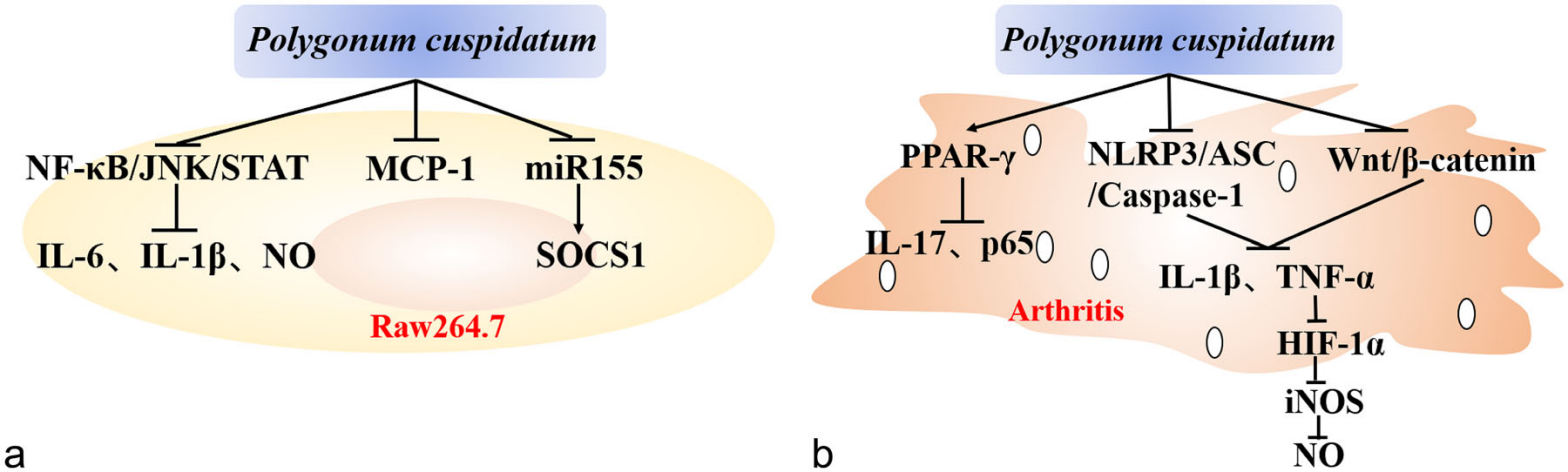
The main mechanism of anti-inflammatory effect of *Polygonum cuspidatum.* (a) In Raw 264.7 cells, the extract of *Polygonum cuspidatum* can decrease the expression of MCP-1, miR155 and the pathway of NF-κB/JNK/STAT directly, and also suppress the expression of IL-1β, IL-6 and NO, as well increase the expression of SOCS1. (b) The extract of *Polygonum cuspidatum* can also treat arthritis in different pathways. It can increase the expression of PPAR-γ, and then the IL-17 as well as p65 are inhibited. In addition, whether the NLRP3/ASC/Caspase-1 or the Wnt/β-catenin pathway can be activated by this plant, their downstream factors, such as IL-1β, TNF-α, HIF-1α, iNOS and NO are induced.

Ethanol extract of PC (100, 200, 400 mg/kg) could prevent colon length shortening and tissue damage, and reduce the levels of inflammatory cytokines in serum of ulcerative colitis mice including interleukin 1β (IL-1β), IL-6, and tumor necrosis factor α (TNF-α). Ethanol extract of PC exerted the above therapeutic effect by regulating nuclear factor kappa-light-chain-enhancer of activated B cells (NF-κB) signal pathway. Researchers discovered that polydatin, resveratrol, and emodin were the primary anti-inflammatory phytochemical components in PC ethanol extract (Liu et al. 2018). Emodin-8-*O*-β-d-glucoside (50, 100, 200 μmol/L), derived from the alcohol extract of PC, could reduce the lipopolysaccharide (LPS) induced inflammation of murine macrophage cell. The above study showed that emodin-8-*O*-β-d-glucoside had significant inhibitory effects on IL-6, IL-1β, and monocyte chemotactic protein-1 (MCP-1) (Li, Yu et al. 2019). Resveratrol and polydatin in PC could also inhibit the releasing of IL-6 and nitric oxide (NO) in the murine macrophage cell inflammatory model in another study, and this effect was related to the suppression on NF-κB and Janus kinase/signal transducer and activator of transcription (STAT) signaling pathway (Ma et al. 2015; Sun et al. 2015). Meanwhile, resveratrol could reduce inflammation by downregulating miR-155 and suppressing of cytokine signaling 1 (SOCS1) (Ma et al. 2017; Figure 1(a)).

*In vitro*, polydatin (15 μg/mL) could reduce the level of IL-1β and TNF-α in human monocytic cells gouty inflammation model and inhibit the expression of pro-inflammatory proteins including toll-like receptor 2 (TLR2), TLR4, and NF-κB (Zhu et al. 2017). In addition, polydatin (20 μmol/L) could also restrain the expression and secretion of MCP-1 in preadipocytes, then inhibited the proliferation and differentiation of preadipocytes. These results indicated that polydatin might treat obesity by regulating the inflammatory (Zheng et al. 2017). Li, Maimai et al. (2019) revealed that polydatin (20, 40, 80 mg/kg) could diminish the infiltration of inflammatory cells in the uterine tissue of endometritis mouse. The protective effect of polydatin might associated with the inhibitory effect on the expression of TNF-α, IL-1β, and IL-6, then suppressed the activation of NF-κB (Li, Maimai et al. 2019). In the mouse mastitis model induced by *Staphylococcus*, polydatin (15, 30, 45 mg/kg) could suppress the activation of the p38 mitogen-activated protein kinase (MAPK)/NF-κB signal pathway, thereby inhibiting the inflammation in breast tissue and reducing tissue damage (Jiang et al. 2017).

*In vivo* experiments showed that PC possessed the potential to treat arthritis (Figure 1(b)). PC could improve synovitis injury in collagen-induced arthritis (CIA) rats by regulating the peroxisome proliferator-activated receptor γ (PPARγ)/NF-κB signal pathway (Yang et al. 2019). In acute gouty arthritis (AGA) mouse model, ethanol extract of PC (90, 180, 360 mg/kg) could inhibit the production of IL-1β and TNF-α in the ankle cavity in a dose-dependent manner. Its mechanism might be ethanol extract of PC could regulate the expression of nucleotide-binding oligomerization domain-like receptor protein 3 (NLRP3)/apoptosis-associated speck-like protein (ASC)/caspase-1 signaling at gene and protein levels (Ma et al. 2019). Researchers found that the crude extract of PC (65, 130, 260 mg/kg) could also improve synovitis symptoms and reduce acute ankle joint swelling in AGA rats. This study further found that this therapeutic effect might be attributed to stilbene and anthraquinone in the crude extract of PC (Ren et al. 2016). In rats with rheumatoid arthritis (RA), polydatin (40, 80, 160 mg/kg) was found to reduce blood levels of TNF- and IL-1 and reduce joint inflammation. This anti-inflammatory effect of polydatin was likely to involve the inhibition of the Wnt/β-catenin signaling pathway (Zeng et al. 2018). In the CIA model, emodin could also alleviate RA by inhibiting synovium inflammation of the knee joint and promoting neovascularization. This effect might be related to emodin could suppress the TNF-α-hypoxia-inducible factor 1α (HIF-1α)-inducible nitric oxide synthase (iNOs)-NO signaling pathway (Wang, Yang et al. 2015; Pan, Wang et al. 2019). In addition, emodin could also alleviate the symptoms of RA rats by up-regulating Bax and Bcl-2 expression (Jin et al. 2018). 8-*O*-β-Glucopyranoside, another quinone compound extracted from the root of PC was found to significantly inhibit the proliferation of fibroblast-like synoviocyte and improve the foot swelling of CIA rats, therefore having therapeutic effects on arthritis (Geng et al. 2018).

The combination of PC and other drugs also showed remarkable anti-inflammatory effects. The combination of PC and *Cinnamomum cassia* Presl (Lauraceae) (Guizhi) alleviated symptoms and played a therapeutic role in rats with AGA. These two Chinese herbs could decrease the expression of pro-inflammatory genes including TLR2, TLR4, and myeloid differentiation factor 88 (MyD88), and reduce the level of IL-1β in the blood of AGA rats (Gu et al. 2015; Cheng and Jiang 2017). Zhang, Wang et al. (2015) found that PC ointment, an external medicine made in China, could reduce tissue inflammation caused by calcium extravasation. The effect was better than the classical medicine of magnesium sulfate (Zhang, Wang et al. 2015). Studies on the anti-inflammatory effects of PC and its extracts are listed in Table 1.

**Table 1.** Anti-inflammatory effects of PC and its compounds.

Pharmacological effects	Mechanism	Extracts/ compounds	Minimal active concentration/dose	Model	Reference
Anti-inflammatory activity	Prevented colon shortening and tissue damage; reduced levels of IL-1β, IL-6, and TNF-α	Ethanol extract	100 mg/kg/day, p.o., for 8 days	Dextran sulfate sodium mice established Ulcerative colitis mice	Liu et al. 2018
	Decreased the levels of IL-6, IL-1β, and MCP-1	Emodin-8-*O*-β-d-glucoside	50 μmol/L	Lipopolysaccharide induced inflammatory model of peritoneal macrophages (RAW264.7 murine macrophages)	Li, Yu et al. 2019
	Decreased the levels of NO and IL-6	Resveratrol	1 μmol/L	Lipopolysaccharide induced inflammatory model of peritoneal macrophages (RAW264.7 murine macrophages)	Ma et al. 2015
	Inhibited the release of IL-6 and NO	Polydatin	100 μg/mL	Lipopolysaccharide induced inflammatory model of peritoneal macrophages (RAW264.7 murine macrophages)	Sun et al. 2015
	Suppressed the expression of IL-6 and TNF-α; inhibited JAK/STAT and MAPK signaling pathway	Resveratrol	1 μg/mL	Lipopolysaccharide induced inflammatory model of peritoneal macrophages (RAW264.7 murine macrophages)	Ma et al. 2017
	Decreased levels of IL-1β, TNF-α; inhibited expression of TLR2, TLR4, and NF-κB	Polydatin	15 μg/mL	Monosodium urate induced THP-1 gouty inflammation model	Zhu et al. 2017
	Inhibited expression and secretion of MCP-1	Polydatin	20 μmol/L	3T3-L1 preadipocytes	Zheng et al. 2017
	Inhibited the expression of TNF -α, IL-1 β, IL-6, and NF-κB activation	Polydatin	20 mg/kg, i.p.	Lipopolysaccharide-induced endometritis in BALB/c mice	Li, Maimai et al. 2019
Anti-inflammatory activity	Suppressed TLR2 expression and p38 MAPK, NF-κB phosphorylation	Polydatin	45 mg/kg, i.p.	Staphylococcus aureus-induced mastitis in BALB/c mice	Jiang et al. 2017
	Reduced inflammatory cell infiltration, promoted the expression of IL-17, and inhibited p65	PC	4 g/kg/day, i.g. for 12 weeks	Bovine collagen II-induced rheumatoid arthritis in SD rats	Yang et al. 2019
	Reduced levels of IL-1β, IL-6, and TNF-α in joint synovium and inhibited NLRP3/ASC/caspase-1 pathway	Ethanolic extract	90 mg/kg/day, i.g., for 6 days	Sodium urate crystals induced acute gouty arthritis in C57BL/6 mice	Ma et al. 2019
	Reduced swelling degree and UA levels	Extract (containing 56.14% anthraquinones and stilbene)	65 mg/kg/day, i.g., for 14 days	Uric acid sodium solution induced gouty arthritis in SD rats	Ren et al. 2016
	Reduced arthritis scores and downregulated Wnt/β-catenin signaling pathway	Polydatin	40 mg/kg/day, i.g., for 28 days	Complete Freund’s adjuvant induced rheumatoid arthritis in SD rats	Zeng et al. 2018
	Relieved inflammation of synovium and promoted angiogenesis	Emodin	0.8 mg/kg, i.g., for 28 days	Bovine collagen II and incomplete Freund Adjuvant induced rheumatoid arthritis in Wistar rats	Wang, Yang et al. 2015
Anti-inflammatory activity	Inhibited TNF-α/HIF-1α/iNOS/NO signaling pathway	Emodin	40 mg/kg/day, i.g., for 20 days	Bovine collagen II and incomplete Freund Adjuvant induced rheumatoid arthritis in Wistar rats	Pan, Wang et al. 2019
	Upregulated Bax mRNA expression and downregulated bcl-2 mRNA expression	Emodin	40 mg/kg/day, i.g., for 20 days	Bovine collagen II and incomplete Freund Adjuvant induced rheumatoid arthritis in Wistar rats Wistar rats	Jin et al. 2018
	Inhibited cell proliferation and TGF-β, NF-κB/MAPK signaling pathway	Physcion8-*O*-β glucopyranoside	IC50 = 49.76 µg/ml	MH7A RA‑derived fibroblast‑like synoviocyte cell	Geng et al. 2018
	Decreased paw swelling and arthritis indices and decreased levels of TNF-α, IL-1β, and IL-6		20 mg/kg	Collagen‑induced arthritis (CIA) rats	
	Decreased the level of IL-1β and suppressed the expression of TLR2, TLR4, and MyD88	PC combinated with *Ramula* *Cinnamomi* （Guizhi）	3.5 g/kg/day, i.g., for 7 days	Monosodium urate induced Acute Gouty Arthritis in SD rats	Gu et al. 2015
	Suppressed the expression TLR2, TLR4	PC combinated with *Artemisia Herba artemisiae* (Yinchen)	1:3 (10 g/kg), i.g., for 10 days	Monosodium urate induced Acute Gouty Arthritis in Wistar rats	Cheng and Jiang 2017
	Relieved inflammatory cell infiltration, edema, and necrosis	PC cream (containing 230 g of knotweed powder)	external use, for 5 days	Calcium extravasation injure model in New Zealand rabbits	Zhang, Wang et al. 2015

### Antioxidant effect

The radical scavenging capacity and oxygen radical absorbance capacity assays indicated that the leaf of PC had the strongest antioxidant capacity, followed by the root and stem (Lachowicz and Oszmiański 2019). The supercritical carbon dioxide liquid extract of PC (10, 20, 50, 100, 250 mg/mL) could scavenge 1,1-diphenyl-2-picrylhydrazyl radical in a concentration-dependent manner. When the concentration was raised to 250 mg/mL, this PC extract had a remarkable capacity for ferric reduction (Lee et al. 2015). *In vitro* experiments revealed that 70% ethanol, ethyl acetate, and butanol extracts of PC strongly inhibited the production of reactive oxygen species by the enzyme xanthine oxidase (Sun, Zhao et al. 2014; Li et al. 2015).

**Figure 2.**
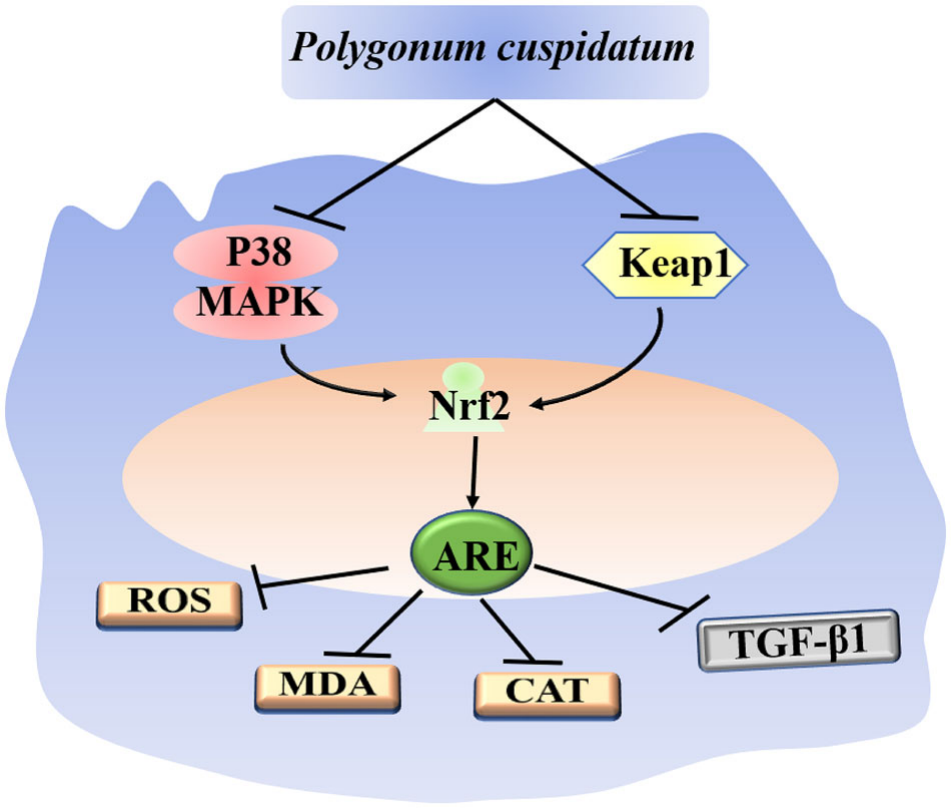
The main mechanism of the antioxidant effect of *Polygonum cuspidatum* Sieb*. et* Zucc. The component of this plant can inhibit the expression of p38 MAPK and Keap1, then Nrf2 is upregulated, which will activate the ARE gene, further induce the expression of ROS, MDA, CAT and TGF-β1.

*In vitro* experiments showed that polydatin (50, 100 μmol/L) could prevent the apoptosis of human umbilical vein endothelial cells (HUVEC) induced by methylglyoxal *via* inhibiting oxidative stress and maintaining mitochondrial function (Pang et al. 2017). In an adriamycin-induced oxidative stress cardiomyopathy rat model, both polydatin and resveratrol (200 μmol/kg) could significantly promote the activities of total superoxide dismutase (T-SOD), catalase (CAT), and glutathione peroxidase (GSH-PX) in plasma and increase the content of GSH in myocardial tissue. These enzymes can prevent excessive levels of reactive oxygen species (ROS) and oxidative stress response in the body (Wang, Gao et al. 2015). Polydatin (0.2 g/kg) could also increase the level of SOD in the serum of atherosclerotic mice, ultimately improving the morphology of atherosclerotic vascular tissue and relieving lipid deposition (Hu et al. 2016). In the ovalbumin-induced asthmatic mice model, polydatin could increase the activity of SOD and CAT in bronchoalveolar lavage fluid and decrease the content of ROS and malondialdehyde (MDA). The mechanism might be related to activating the p38 MAPK/nuclear factor E2-related factor 2 (Nrf2) signal pathway (Zhao, Jiang et al. 2018; Zeng et al. 2019). Researchers found that polydatin could also reduce the ROS content in the fat tissue of the retrobulbar of Graves’ orbitopathy mice model by stimulating the Kelch like-ECH-associated protein 1 (Keap1)/Nrf2 pathway in orbital fibroblasts (Li et al. 2020). The stimulation of PC on Keap1/Nrf2 could also reduce the ROS level in the liver tissue of rats with fructose-induced liver injury and inhibit fatty liver degeneration (Zhao, Yu et al. 2018). Figure 2 depicts the primary mechanisms of PC’s antioxidant effect. Studies on the antioxidant effects of PC and its extracts are listed in Table 2.

**Table 2.** Antioxidant effects of PC and its compounds.

Pharmacological effects	Mechanism	Extracts/compounds	Minimal active concentration/dose	Model	Reference
Antioxidant activity	Promote radical scavenging	Supercritical carbon dioxide fluid extraction	10 mg/mL	DPPH assay	Lee et al. 2015
	Inhibited xanthine oxidase	70% Ethanol extracts	IC_50_ = 2.06 mg/mL	HPLC method for *in vitro* screening	Sun, Zhao et al. 2014
	Inhibited xanthine oxidase	Ethyl acetate and butanol extractions	K_m_ = 40 μg/mL, 100 μg/mL	HPLC method for *in vitro* screening	Li et al. 2015
	Inhibited methylglyoxal-induced cell apoptosis and activated Akt pathway	Polydatin	50 μmol/L	Methylglyoxal-induced HUVEC apoptosis	Pang et al. 2017
	Promote the activities of T-SOD, CAT, and GSH-Px in plasma; increased the GSH in myocardial tissue	Polydatin and resveratrol	200 μmol/kg, i.g., for 15 days	Doxorubicin induced oxidation model in SD rats	Wang, Gao et al. 2015
	Reduced ROS level and promoted SOD activity	Polydatin	0. 2 g/kg/day, i.g., for 28 days	High fat diet induced ApoE^-/-^ mice model of coronary atherosclerosis	Hu et al. 2016
	Activated p38 MAPK/Nrf2 signaling pathway	Polydatin	30 mg/kg/day, i.p., for 7 days	Ovalbumin-induced Asthma BALB/c mice	Zhao, Jiang et al. 2018
	Inhibited activity of ROS, TGF-β1, and Nrf2	Polydatin	100 mg/kg	Ovalbumin-induced Asthma BALB/c mice	Zeng et al. 2019
Antioxidant activity	Reduced ROS level and activated Keap1/Nrf2/ARE pathway	Polydatin	10 μmol/L	H2O2-induced oxidative stress in orbital fibroblasts	Li et al. 2020
			50 mg/kg/day, i.g., for 4 weeks	Adenovirus expressing the thyroid-stimulating hormone receptor (TSHR) A-subunit (Ad-TSHR289) induced Graves’ orbitopathy mice	
	Reduced ROS level, inhibited Keap1 and activate Nrf2 pathway	Polydatin	40 μmol/L	Fructose induced BRL-3A and HepG2 cells injury	Zhao, Yu et al. 2018
			7.5 mg/kg, i.g., for 7 weeks	Fructose induced liver oxidative stress, inflammation, and lipid deposition in SD rats	

### Anticancer effect

*In vivo* experiments showed that PC (300 mg/kg) could inhibit osteosarcoma cell growth. PC could initiate apoptosis and S-phase cell cycle arrest in osteosarcoma cells through impeding protein kinase B (AKT)/extracellular signal-regulated protein kinase (ERK)/epidermal growth factor receptor (EGFR) pathway (Zhao, Pan et al. 2022). Crude extract of PC (25, 50, 100, 200, 400 μg/mL) could inhibit the activity of human breast cancer cells and ovarian cancer cells, as well as promote cancer cell apoptosis in a dose and time-dependent manner, with IC_50_ (50% of the maximum inhibitory concentration) values of 31.18 ± 1.95 μg/mL and 28.12 ± 1.07 μg/mL, respectively (Pan, Shi et al. 2019). PC ethanol extract (50, 100, 150, 200 μg/mL) could also suppress the cell viability of cisplatin-resistant human oral cancer cells *in vitro* and promote cancer cell apoptosis through the endogenous pathway (caspase-3/9) (Wang, Horng et al. 2019). *In vitro* experiments showed that when the concentration was higher than 25 mg/mL, supercritical carbon dioxide liquid extract of PC could significantly inhibit tyrosinase activity in melanoma cells. This PC extract might lower the amount of melanin in melanoma cells when the concentration reached 50 mg/mL. Moreover, this PC extract could also induce the release of TNF-α from human monocytic after 48 h of culture. TNF-α is a kind of inflammatory/killing cytokine, and such immune stimulation might promote PC extract’s antitumor activity (Lee et al. 2015).

*In vivo* experiments showed that polydatin (50 mg/kg) could restrain the growth of human laryngeal cancer cells transplanted into nude mice and diminish the tumor weight by 40%. Researchers found that polydatin could inhibit the proliferation of cancer cells by suppressing the activation of the platelet-derived growth factor (PDGF)/AKT signaling pathway in a dose-dependent manner (Li et al. 2017). Polydatin could also induce the S-phase arrest of human acute monocytic leukemia cell line THP-1 dose-dependently and inhibit the proliferation of cancer cells. The mechanism might regulate Bcl-2, Bax, cyclin A, and cyclin D1 expression (Wang, Luo et al. 2016). Moreover, polydatin could also inhibit the proliferation of leukemia cell line K562 by regulating the AKT/mammalian target of rapamycin (mTOR)/P70S6K signaling pathway (Luo et al. 2016). In addition to these cancer cells, *in vitro* experiments also showed that polydatin could inhibit the growth of cervical cancer (Pan et al. 2017), lung cancer (Sun and Ye 2019), and breast cancer (Chen et al. 2017; Feng et al. 2019).

Resveratrol also showed a prominent anticancer effect. It could down-regulate Bcl-2 protein and up-regulate the expression of Bax protein, and induce human liver carcinoma cells into phase S and promote apoptosis (Gu et al. 2014). In addition, resveratrol could also inhibit the growth of the human gastric adenocarcinoma cells and arrest the cancer cells at the G0/G1 stage (Jing et al. 2016). Emodin (20 μmol/L) could inhibit the expression of transforming growth factor β2 (TGF-β2) and reduce the cell viability and colony formation of human ovarian cancer cells. This effect of emodin was mediated by activating forkhead box protein D3 (FoxD3) and miR-199a (Song et al. 2018). Rhein, an anthraquinone compound that can be isolated from PC, was found to suppress tumors *in vitro* by interfering with Pin1/c-Jun interaction (Cho et al. 2017). Moreover, rhein could also reverse the adriamycin resistance in the hepatoma cell line in a dose-dependent manner and increase adriamycin in the cells. Moreover, 20 μmol/L rhein could enhance the sensitivity to adriamycin of the hepatoma cell line by 7.24 times (Wu, Cao et al. 2019). These studies may provide a new direction for the treatment of drug resistance in cancer cells.

Resveratrol-4-*O*-d-(2′-galloyl)-glucopyranoside is one of the active compounds isolated from PC. Xie et al. (2014) revealed that this compound could inhibit the activity of human hepatoma cells and slow tumor growth in mice transplanted with hepatoma cells. The mechanisms included the regulation of the c-Jun-N-terminal kinase (JNK)/ERK signaling pathway, and this effect was dose- and time-dependent (Xie et al. 2014). Another active compound in the ethyl acetate (EtOAc) extract of PC, 2-ethoxystypandron, could also restrain the cell growth of human hepatoma cells in a dose-dependent manner. This effect of 2-ethoxystypandron was linked to its inhibition of STAT3 signaling and the induction of hepatoma cell cycle arrest (Li, Zhang et al. 2019). 2-Methoxystypandron, another EtOAc extract of the PC roots was found to be an inhibitor of JAK2 and IκB kinase. 2-Methoxystypandron could also inhibit the activation of STAT3 induced by IL-6 and suppress the growth of cervical cancer cells. Even a concentration of 10 μmol/L could completely inhibit the growth of cancer cells (Kuang et al. 2014). 2-Methoxy-6-acetyl-7-methyljuglone, the active component of PC, could significantly reduce the proliferation of 16 types of cancer cell lines *in vitro*, and its IC_50_ value was less than 5.5 μmol/L (Sun et al. 2016). Studies on the anticancer effects of PC and its extracts are listed in Table 3.

**Table 3.** Anticancer effects of PC and its compounds.

Pharmacological effects	Mechanism	Extracts/compounds	Minimal active concentration/dose	Cancer	Reference
Anticancer activity	Initiated apoptosis and S-phase cell cycle arrest through impeding Akt/ ERK/EGFR pathways	PC dissolved in saline	300 mg/kg	Osteosarcoma	Zhao, Pan et al. 2022
	Inhibited the activity of human breast cancer and promoted apoptosis	Crude extract	IC_50_ = 31.18 ± 1.95 μg/mL	Breast cancer	Pan, Shi et al. 2019
			IC_50_ = 28.12 ± 1.07 μg/mL	Ovarian cancer	
	Anti-human oral cancer, stimulated caspase-9 and -3 activities	Ethanol extract	50 μg/mL	Oral cancer	Wang, Horng et al. 2019
	Anti-laryngeal cancer and inhibited the PDGF/AKT signaling pathway	Polydatin	2 μmol/L	Laryngeal cancer	Li et al. 2017
	Reduced mean tumor volume		50 mg/kg		
	Anti-leukemia, induced S-phase cell cycle arrest, and upregulated cyclin D1 and Bcl-2	Polydatin	IC_50_ (48 h) = 50 μmol/L	Acute leukemia	Wang, Luo et al. 2016
	Anti-leukemia and inhibited Akt/mTOR/p70S6K signaling pathway	Polydatin	IC_50_ (24 h) = 80 μmol/L	Acute leukemia	Luo et al. 2016
	Anti-cervical cancer and inhibited PI3K/AKT/mTOR pathway	Polydatin	50 μmol/L	Cervical cancer	Pan et al. 2017
	Anti-hepatoma and inhibited AKT/NF-κB pathway	Polydatin	IC_50_ (72 h) = 34.89 mg/L	Lung cancer	Sun and Ye 2019
	Inhibited the proliferation of human breast cancer and downregulated VEGF and MMP-9	Polydatin	0.2 μmol/L	Breast cancer	Chen et al. 2017
Anticancer activity	Induced phase S cell cycle arrest and downregulated CREB and cyclin D1	Polydatin	1.5 μmol/L	Breast cancer	Feng et al. 2019
	Inhibited the proliferation of cells, downregulated the expression of Bcl-2 and upregulated Bax	Resveratrol	25 μmol/L	Liver cancer	Gu et al. 2014
	Induced cell blockage at phase S	Resveratrol	12.5 μmol/L	Liver cancer	Gu et al. 2015
	Decreased the survival rate of cells and rested the cells at the G0/G1 phase	Resveratrol	IC_50_ (24 h) = 127 μmol/L	Gastric cancer	Jing et al. 2016
	Anti-ovarian cancer, promoted FOXD3 expression, activated miR-199a, and suppresses the expression of TGF-β2	Emodin	20 μmol/L	Ovarian cancer	Song et al. 2018
	Increased accumulation of DOX in SMMC-7721/DOX cells	Rhein	20 μmol/L	Liver cancer	Wu, Cao et al. 2019
	Inhibited human hepatoma cells activity	Resveratrol-4-*O*-d-(2′-galloyl)-glucopyranoside	2.5 μmol/L	Liver cancer	Xie et al. 2014
	Reduced mean tumor volume and weight in mice		10 mg/kg		
	Anti-hepatoma and inhibited STAT3 signaling	2-Ethoxystypandrone	IC_50_ = 3.69 ± 0.51 μmol/L	Liver cancer	Li, Zhang et al. 2019
			IC_50_ = 5.58 ± 0.89 μmol/L		
	Induced death of tumor cells and inhibited STAT3 and NF-κB pathways	2-Methoxystypandrone	10 μmol/L	Cervical cancer, Breast Cancer, Glioma, Ovarian cancer, Prostate cancer, Lung cancer	Kuang et al. 2014
	Induced multiple forms of cell death in cancer cells and activated JNK/iNOS/NO pathways	2-Methoxy-6-acetyl-7-methyl-juglone	IC_50_ < 5.5 μmol/L	Lung cancer, Melanoma, Breast cancer	Sun et al. 2016

### Neuroprotection effect

The main components of PC have shown significant neuroprotective effects in many neurological diseases. Polynapstilbene B, resveratrol, (-)-epicatechin, procyanidin B 2, 3-*O*-gallate, and 2′-*O*-galloyl-peceid were isolated from PC. These components could significantly relieve the damage of PC12 cells caused by rotenone at 10 μmol/L *in vitro*, among which resveratrol had the most potent inhibitory effect (71.7%) (Liu et al. 2015). Studies observed that physcion 8-*O*-β-glucopyranoside, isolated from PC, could alleviate the symptoms of dementia rats and reduce the increase in escape time of dementia rats in Morris water maze by 44.8% (Xu et al. 2015).

The chronic and unpredictable mild stress (CUMS) rat model was established to investigate the effect of resveratrol on depression. Long-term use of resveratrol could significantly prevent behavioral changes induced by CUMS, such as spatial learning and memory disorders (Liu, Zhang et al. 2014). In addition, resveratrol could up-regulate the cAMP-response element-binding protein (CREB) and brain derived neurotrophic factor (BDNF) and modulate the mRNA levels of Bcl-2 and Bax in the hippocampus of CUMS rats (Wang, Xie et al. 2016; Shen et al. 2018). Resveratrol also had an antidepressant effect by regulating serum corticosterone levels. When trans-resveratrol was combined with piperine, a bioavailability enhancer, the minimum effective dose of trans-resveratrol could be reduced to 20 mg/kg (Liu, Xie et al. 2014; Xu et al. 2016). In addition to alleviating symptoms of depression, resveratrol (10, 100 nmol/L) significantly reduced hypoxia-induced degradation of IκB-α, phosphorylation of p65 NF-κB protein, ERK1/2, and JNK, thereby inhibiting microglial activation (Zhang, Yuan et al. 2015).

### Hepatoprotective effect

Continuous gavage of PC water extract (80, 160 mg/kg) for 11 weeks could decrease liver lipid accumulation in fructose-fed rats with metabolic syndrome by targeting the Keap1/Nrf2 pathway (Zhao, Chen et al. 2022). In addition, polydatin (50, 100 mg/kg) could also reduce alanine transaminase and aspartate aminotransferase in serum of mice with alcohol induced hepatic injury (Koneru et al. 2017). Furthermore, polydatin (6.25 mg/mL) could alleviate hepatic steatosis in zebrafish larvae induced by ethanol. This effect of polydatin might correlate with the improvement of ethanol and fat metabolism, inhibition of oxidative stress and DNA damage (Lai et al. 2018).

### Cardioprotective effect

Ding W et al. (2014) have found that polydatin is essential in preventing pressure overload-induced cardiac hypertrophy and heart failure. The mechanism might be polydatin could inhibit the Ca^2+^-calcineurin pathway without affecting myocardial contractility (Ding W et al. 2014). Furthermore, trans-polydatin could also decrease the expression of angiotensin, inhibit the activity of renin and angiotensin-converting enzyme, and protect against myocardial ischemia injury (Ming et al. 2017). This suggested that PC might be a new cardiac protective drug.

### Blood vessel protective effect

The extracts of PC root (100, 350 mg/kg) could inhibit the increase of retinal vascular permeability in diabetic rats, suggesting that oral administration might help to suppress the development of retinopathy in diabetic patients (Sohn et al. 2016). *In vitro*, it was shown that polydatin (1, 3, 10 μmol/L) could restore abnormal vasodilation induced by high glucose levels in a dose-dependent manner. When the concentration of polydatin was 10 μmol/L, it could recover vascular endothelial function to the same level as the normal glucose group. The effect of polydatin was related to activating the PPAR protein-NO pathway (Wu et al. 2015). Researchers discovered that quercetin (0.1, 0.5, 1 μmol/L) could protect human brain microvascular endothelial cells injured from hypoxia and reoxygenation. Regulating the Keap1/Nrf2 pathway and endoplasmic reticulum stress might be important for the protective effect of quercetin. Such effects suggest that quercetin may protect small vessels and thus be a potential treatment for cerebral small vessel disease (Li et al. 2021).

### Antiviral effect

Procyanidin c-13,3′,3′′-tri-*O*-gallate was isolated from the ethanol extract of PC and could activate the transcription of HIV-1 in a concentration and time-dependent manner. This suggested that procyanidin c-13,3′,3′′-tri-*O*-gallate could be combined with highly active antiretroviral therapy to eliminate inactive potential HIV (Wang, Yang et al. 2015). The aqueous extract of PC, with resveratrol and emodin as the most effective active compounds, was found to inhibit the replication of the H1N1 influenza virus *in vitro*, with an IC_50_ value of 312 g/mL (Lin et al. 2015). Both the ethanol and aqueous extracts of PC were able to inhibit 3-chymotrypsin-like (3CL) protease and prevent the interaction between spike-protein and angiotensin-converting enzyme II, which in turn prevented the entry of SARS-CoV-2 wild-type and omicron pseudotyped viruses into intact zebrafish larvae. The researchers further found that among the 9 major phytochemical constituents in these PC extracts, only gallic acid significantly inhibited viral entry into HEK293T cells in a dose-dependent manner, with an IC_50_ value of 23.5 μmol/L (Lin et al. 2022). Other studies have shown that EtOAc extract from the root of PC (12.5 μg/mL) could suppress the expression of Epstein-Barr virus lytic proteins and transcriptional genes, and the transcriptional inhibition rates of lytic genes BRLF1 and BZLF1 are 95.29% and 95.31%, respectively (Yiu et al. 2014).

Orthoquin, a natural product extracted from the root of PC, could react with oxygen to produce singlet oxygen and short-lived reactive oxygen species. These oxygen species can damage large molecules such as proteins, lipids, and nucleic acids near the virus, then inhibit viral replication. Orthoquin could inhibit herpes simplex virus infection in a light-dependent manner (Monjo et al. 2018). Resveratrol (30 g/mL) and polydatin (200 g/mL) were both shown to have anti-human enterovirus-71 properties *in vitro* and to be able to protect rhabdosarcoma cells. Compared to polydatin, resveratrol exhibited a stronger antiviral effect (Zhang, Li et al. 2015).

### Antibacterial and antifungal effects

Different extracts from PC have extraordinary bacteriostatic effects. The methanol extract of PC root could significantly suppress the activity of bacterial neuraminidase and alleviate the symptoms of the host. In particular, the active ingredient emodin-1-*O*-β-d-glucopyranoside in PC extract demonstrated a robust inhibitory effect on activity of bacterial neuraminidase at a low concentration (IC_50_ = 0.43 μmol/L) (Uddin et al. 2016). The ethanol extract of PC had inhibitory effects on *Bacillus subtilis*, *Staphylococcus aureus*, and *Pseudomonas aeruginosa*, with minimum inhibitory concentration (MIC) values of 100, 50, and 100 μg/mL. This effect of PC extract suggested that it can treat bacterial infection caused by snakebite (Liu, Nielsen et al. 2014). The ethyl ether fraction of PC also showed a broad antimicrobial spectrum against the tested clinical drug-resistant isolates, with the MIC between 0.2 ∼ 1.63 mg/mL, which was 3 to 10 times more effective than crude PC extract. The ethyl ether fraction of PC at 2 times the MIC could completely kill 3 × 10^5^ CFU/mL *Staphylococcus aureus* within 1 h (Su et al. 2015). In addition, the ethyl ether fraction of PC had anti-methicillin-resistant *Staphylococcus aureus* activity, as it could destroy the integrity of bacterial cell walls and cell membranes. It was found that emodin (32 g/mL) was the main component of ethyl ether fraction of PC to reduce the activities of the methicillin-resistant *Staphylococcus aureus*. Emodin could inhibit the expression of biofilm-related genes, reduce the release of extracellular DNA, and thus inhibit the formation of *Staphylococcus aureus* biofilm in a dose-dependent manner (Cao et al. 2015). Moreover, the extracts of PC could also be used as a chemical stabilizer that binds to the surface of silver nanoparticles to enhance their antibacterial effect (Sun, Qu et al. 2014).

Studies have shown that PC had different antifungal activities against superficial fungi: *Trichophyton rubrum* (MIC = 50 μg/mL), *Trichophyton mentagrophytes* (MIC = 100 μg/mL), *Microsporum canis* (MIC = 50 μg/mL), *Epidermophyton floccosum* (MIC = 50 μg/mL), *Trichophyto schoenleinii* (MIC = 50 μg/mL), *Microsporum gypseum* (MIC = 100 μg/mL), *Trichophyton tonsurans* (MIC = 50 μg/mL), and *Trichophyton violaceum* (MIC = 50 μg/mL) (Yang et al. 2015).

### Other pharmacological effects

The water extract of PC (100, 250 mg/kg) significantly reduced the corneal irregular score and increased the volume of tears after extra orbital lacrimal gland resection in dry-eye rats. Park et al. (2018) have proved that this effect of PC extract might be related to the increased expression of mucin-4 and the inhibition of oxidative stress and inflammation (Park et al. 2018). PC and its extracts also have a strong regulatory effect on carbohydrate and lipid metabolism. The α-glucosidase and protein-tyrosine phosphatase 1B (PTP1B) were essential in insulin metabolism. *In vitro*, it was found that the crude EtOAc extract of PC could inhibit the activity of α-glucosidase and PTP1B, with IC_50_ values of 8.33 ± 1.42 and 16.21 ± 0.38 μg/mL, respectively (Zhao et al. 2017). Ethanol extract from the root of PC (100, 350 mg/kg) could inhibit the expansion and proliferation of glomerular mesangial matrix in diabetic rats, thus preventing diabetic nephropathy (Sohn et al. 2014).

In addition, resveratrol (20, 50, 100 μmol/L) could up-regulate the expression of type II collagen in superficial chondrocytes and middle chondrocytes, indicating that it could be used in the treatment of arthritis (Maepa et al. 2016). In a clinical test, the plant was found to have the ability to inhibit platelet aggregation. When healthy subjects took a supplement (80 mL) containing 10% resveratrol, the platelet aggregation induced by the platelet-activating factor could be suppressed significantly (Gavriil et al. 2019). The inhibitory effect of polydatin on angiogenesis allowed it to be used to treat angiogenesis-related diseases, including retinopathy, rheumatoid arthritis, and psoriasis (Hu et al. 2019). Emodin is considered as a potential therapeutic drug for lung cancer induced-cachexia, because feeding with emodin-enriched PC extract (2% of feed supplement) could increase the weight and reduce gastrocnemius muscle atrophy of A549 tumor-bearing BALB/c-nu mice. *In vivo* and *in vitro* mechanism research showed that emodin in this PC extract could inhibited transcription factor 4 (TCF4)–TWIST1 (a bHLH-domain-containing transcription factor) interaction, then suppress parathyroid hormone-related protein (PTHrP) expression (Fang et al. 2022).

### Summary of pharmacologic effects

PC has been prescribed for medicinal purposes for thousands of years in China. In addition to anti-inflammatory, antioxidative, anticancer, and neuroprotective properties, PC and its phytochemical constituents had protective effects on the heart, kidney, liver, and other organs. PC can be a valuable alternative to various diseases, including inflammation, cancer, cognitive impairments, depression, fatty liver disease, and diabetes. Identifying the best therapeutic effect of one or more phytochemical constituents of PC and analyzing its mechanisms of action would help with the development of PC-related medicines. In addition, the above studies on the pharmacological activities of PC and its phytochemical constituents were all animal or cell experiments, and the therapeutic effects and safety need to be verified in future clinical studies.

#### Clinical application

This review summarized the dosage and compatibility of PC in prescriptions of past dynasties and found that the effective dosage of PC mainly was between 10 g and 30 g. When the dosage was ≤ 30 g, it primarily removes dampness and jaundice, clears heat, and helps with body detoxification; when the dosage was greater than 10-90 g, it promotes blood circulation and dredged channels. The most commonly drugs compatible to be used with PC supplements were blood-activating and stasis-dissolving drugs, heat-clearing drugs, blood-enriching drugs, Qi-tonic drugs, and water-dampening drugs (Bai et al. 2016), which were used to treat severe moldy sugarcane poisoning, cirrhosis ascites, carotid atherosclerosis, etc. In addition, PC could also be made into a tincture to treat burns or made into an ointment to assist in the treatment of periappendiceal abscess (Liu, Zheng et al. 2014; Xia and Yang 2014; Wang, Yang et al. 2015; Li, Bei et al. 2016; Chen et al. 2018). Compatible drugs and appropriate dosage for different diseases should be carefully selected when clinicians prescribe the medicine.

The information on proprietary Chinese medicines with PC as the main component in the State Drug Administration (https://www.nmpa.gov.cn) was also queried. The results included Huzhangye capsules (approval number: Z20026314), Huzhangfanshi liniments (approval number: Z20025342), Huzhangshangtong tincture (approval number: Z20025395), Compound Huzhang tablets (approval number: Z45022334), Compound *Rhizoma Polygoni Cuspidati* burn oil (approval number: Z10920021), and Compound paracetamol and chlorphenamine maleate capsules (approval number: H13023540). Huzhangye capsules were used to treat dizziness, headaches, and other symptoms related to hypertension (Zhao 2016). It was safe and effective in treating primary hypertension with liver Yang hyperactivity when used in combination with nifedipine. Its antihypertensive mechanism might be related to the protection of vascular endothelial cells (Ding and Gao 2021). Experimental findings also indicated that Huzhangye capsules were more effective in improving the symptoms of benign paroxysmal positional vertigo when combined with manual reduction (Zhang et al. 2020). Compound Huzhang tablets could clear heat and remove phlegm, relieve coughs and asthma; it could also be used to prevent liver damage from psychotropic drugs (Yang and Liu 2014). Compound paracetamol and chlorphenamine maleate capsules, which was made according to the principle of combining Chinese and western medicine to treat cold, contain acetaminophen, chlorpheniramine maleate, and PC. Huzhangshangtong tincture, Huzhangfanshi liniments, and Compound *Rhizoma Polygoni Cuspidati* burn oil are all external drugs. Huzhangshangtong tincture was used to treat traumatic injuries (Liu 2016), and the latter two were used to treat mild water and fire burns (Liu et al. 2012).

#### Pharmacokinetics

Most of the current pharmacokinetic studies on PC focused on its active ingredients. This review discussed the pharmacokinetic studies of four richest ingredients in PC. Resveratrol was characterized by low solubility and high intestinal permeability, with a plasma bioavailability of about 1% after oral administration. Glucuronides and monosulfates were the main metabolites in plasma (Huang et al. 2019; Briskey and Rao 2020; Zhang et al. 2021). After oral administration, resveratrol was swiftly metabolized in the liver and intestine, mostly into sulfate conjugates and glucuronides, and excreted through the urine (Honari et al. 2019). It has also been reported that the above metabolites could be converted back to resveratrol by intestinal microbes (Zhang et al. 2021). Polydatin, a glucose derivative of resveratrol, was mainly metabolized to resveratrol in the small intestine and liver, then metabolized to glucuronidation forms, but polydatin could still be detected in plasma and urine (Lou et al. 2021; Montanari et al. 2021; Sunsong et al. 2021). Animal experiments showed the mutual transformation between polydatin and resveratrol in rats after oral administration of polydatin and resveratrol at the same dosages, respectively (Wang, Gao et al. 2015).

After oral administration of PC, emodin also rapidly underwent phase II metabolism to form its glucuronide. The parent form of emodin was of low concentration in the body, which was only detectable in the liver and the brain (Di et al. 2015; Dong et al. 2016). A study in mice showed that plasma glucuronidated emodin peaked 1 h after intragastric administration of emodin and was eliminated within 12 h. Female mice appeared to metabolize emodin faster than male mice (Sougiannis et al. 2021). Studies have shown that quercetin was present in a conjugated form whose primary form was glycoside in human blood after a single oral dose (Li, Yao et al. 2016). After oral administration of 200 mg of quercetin, the C_max_ and T_max_ were 2.3 ± 1.5 µg/mL and 0.7 ± 0.3 h, respectively (Batiha et al. 2020). Quercetin aglycones were absorbed in the small intestine mainly through passive diffusion and transported by organic anion transport peptides, followed by methylation, vulcanization, and glucuronidation in the small intestine and liver (Li, Yao et al. 2016). After metabolized by the liver, it could enter the circulation or be metabolized by the kidney and finally excreted from urine (Guo and Bruno 2015; Li, Yao et al. 2016).

In general, the primary components of PC were rapidly metabolized by the small intestine and liver after entering the body. More studies should be carried out on improving drug solubility, controlling drug release, preventing drug degradation, changing the means of administration, and preventing metabolism for enhancing the bioavailability of drugs.

## Conclusions and future perspectives

PC has been used in the clinical practice for thousands of years and has extensive pharmacological activities. This review summarized its phytochemical constituents, pharmacological actions, the clinical application, and pharmacokinetics. PC and its main components have a wide range of pharmacological activities and are used for antipyretic, antibacterial, anticancer, cardiovascular and cerebrovascular protection, etc. It could be an effective therapeutic drug for various related diseases, such as arthritis, ulcerative colitis, asthma, and cardiac hypertrophy. PC can exert its therapeutic effects on a variety of systems and pathways since it contains a variety of phytochemical ingredients.

The synergistic therapeutic effect of the main phytochemical constituents in PC and the herb-drug interaction should also be further explored, which can guide the clinical drug use and standardized drug preparation. Many active ingredients that come from the roots of PC have been studied. The phytochemical constituents and pharmacological effects of flowers or leaves have been less studied, which limits the clinical use of PC. In the future, more research on other parts of PC should also be done to expand the understanding of PC.

Pharmacokinetic studies showed that the poor bioavailability of PC and its main phytochemical constituents affected its medicinal properties. Contemporary technology should be used to develop biological preparations of compounds with improved bioavailability and tested them in animals or even humans for early application in the clinical treatment. Although PC has been reported to be nephrotoxic in some cases, its contributions to the clinical treatment are undeniable. With more research being done on PC, its pharmacological effects and safety will be better known, allowing for more widespread therapeutic application of these plants.

## References

[CIT0001] Acquaviva R, Malfa GA, Di Giacomo C. 2021. Plant-based bioactive molecules in improving health and preventing lifestyle diseases. IJMS. 22(6):2991. 33804225 10.3390/ijms22062991 PMC8000372

[CIT0002] Agbabiaka TB, Spencer NH, Khanom S, Goodman C. 2018. Prevalence of drug-herb and drug-supplement interactions in older adults: a cross-sectional survey. Br J Gen Pract. 68(675):e711–e717. 30249608 10.3399/bjgp18X699101 PMC6145997

[CIT0003] Bai Y, Tong R, Li J. 2016. Overview of the effects of different doses and combinations on the efficacy of *Polygonum cuspidatum*. Sieb. et Zucc. China Pharm. 27:105–107. (Chinese).

[CIT0004] Batiha GE-S, Beshbishy AM, Ikram M, Mulla ZS, El-Hack MEA, Taha AE, Algammal AM, Elewa YHA. 2020. The pharmacological activity, biochemical properties, and pharmacokinetics of the major natural polyphenolic flavonoid: quercetin. Foods. 9(3):374. 32210182 10.3390/foods9030374 PMC7143931

[CIT0005] Briskey D, Rao A. 2020. *trans*-Resveratrol oral bioavailability in humans using lipiSperse dispersion technology. Pharmaceutics. 12(12):1190. 33302446 10.3390/pharmaceutics12121190 PMC7763804

[CIT0006] Cao F, Peng W, Li X, Liu M, Li B, Qin R, Jiang W, Cen Y, Pan X, Yan Z, et al. 2015. Emodin is identified as the active component of ether extracts from *Rhizoma Polygoni Cuspidati*, for anti-MRSA activity. Can J Physiol Pharmacol. 93(6):485–493. 25966789 10.1139/cjpp-2014-0465

[CIT0007] Chen Z, He J, Zhou X, Wang M, Wen K. 2018. Observation on the curative effect of *Polygonum cuspidatum* in auxiliary treatment of periappendiceal abscess. Chin J Integr Tradit West Med. 38:755–756. (Chinese).

[CIT0008] Chen S, Tao J, Zhong F, Jiao Y, Xu J, Shen Q, Wang H, Fan S, Zhang Y. 2017. Polydatin down-regulates the phosphorylation level of Creb and induces apoptosis in human breast cancer cell. PLoS One. 12(5):e0176501. 28467448 10.1371/journal.pone.0176501 PMC5415055

[CIT0009] Cheng X, Jiang X. 2017. Effect of different ratios of *Polygonum cuspidatum* Sieb. et Zucc and *Artemisia Herba artemisiae* on acute gouty arthritis rats. Pharmacol Clin Chin Mater Med. 33:102–105. (Chinese).

[CIT0010] Cho JH, Chae JI, Shim JH. 2017. Rhein exhibits antitumorigenic effects by interfering with the interaction between prolyl isomerase Pin1 and c-Jun. Oncol Rep. 37(3):1865–1872. 28184937 10.3892/or.2017.5434

[CIT0011] Di X, Wang X, Di X, Liu Y. 2015. Effect of piperine on the bioavailability and pharmacokinetics of emodin in rats. J Pharm Biomed Anal. 115:144–149. 26201645 10.1016/j.jpba.2015.06.027

[CIT0012] Ding W, Dong M, Deng J, Yan D, Liu Y, Xu T, Liu J. 2014. Polydatin attenuates cardiac hypertrophy through modulation of cardiac Ca^2+^ handling and calcineurin-NFAT signaling pathway. Am J Physiol Heart Circ Physiol. 307(5):H792–802. 25015961 10.1152/ajpheart.00017.2014

[CIT0013] Ding MM, Gao JB. 2021. Clinical study on Huzhangye capsules combined with nifedipine in treatment of primary hypertension with liver Yang hyperactivity. Drugs Clinic. 36:777–781. (Chinese).

[CIT0014] Dong X, Fu J, Yin X, Cao S, Li X, Lin L, Ni J, Huyiligeqi 2016. Emodin: a review of its pharmacology, toxicity and pharmacokinetics. Phytother Res. 30(8):1207–1218. 27188216 10.1002/ptr.5631 PMC7168079

[CIT0015] Fang XQ, Kim YS, Lee YM, Lee M, Lim WJ, Yim WJ, Han MW, Lim JH. 2022. *Polygonum cuspidatum* extract (Pc-Ex) containing emodin suppresses lung cancer-induced cachexia by suppressing TCF4/TWIST1 complex-induced PTHrP expression. Nutrients. 14(7):1508. 35406121 10.3390/nu14071508 PMC9002362

[CIT0016] Feng C, Shao H, Wang Z, Zhu S, Zheng X. 2019. Effects of polydatin on cell proliferation, migration and invasion of breast cell line MDA- MB- 231. Chin J Immunol. 35:1204–1207. +1212. (Chinese).

[CIT0017] Gavriil L, Detopoulou M, Petsini F, Antonopoulou S, Fragopoulou E. 2019. Consumption of plant extract supplement reduces platelet activating factor-induced platelet aggregation and increases platelet activating factor catabolism: a randomised, double-blind and placebo-controlled trial. Br J Nutr. 121(9):982–991. 30940217 10.1017/S0007114519000308

[CIT0018] Geng Q, Wei Q, Wang S, Qi H, Zhu Q, Liu X, Shi X, Wen S. 2018. Physcion 8‑*O*‑β‑glucopyranoside extracted from *Polygonum cuspidatum* exhibits anti‑proliferative and anti‑inflammatory effects on MH7A rheumatoid arthritis‑derived fibroblast‑like synoviocytes through the TGF‑β/MAPK pathway. Int J Mol Med. 42:745–754. 29717774 10.3892/ijmm.2018.3649 PMC6034927

[CIT0019] Gu Z, Huang H, Shi W, Hu X, Han B. 2015. Impacts of herb pair of *Rhizoma Polygoni Cuspidati* and *Ramula Cinnamomi* on acute gouty arthritis. Tradit Chin Drug Res Clin Pharmacol. 26:315–319. (Chinese).

[CIT0020] Gu S, Li M, Zhu K. 2014. Apoptosis of HepG-2 cell induced by resveratrol and its effects on expressions of proteins Bcl-2 and Bax. Chin J Exp Tradit Med Formulae. 20:168–172. (Chinese).

[CIT0021] Guo Y, Bruno RS. 2015. Endogenous and exogenous mediators of quercetin bioavailability. J Nutr Biochem. 26(3):201–210. 25468612 10.1016/j.jnutbio.2014.10.008

[CIT0022] Guo S, Li Y, Su H, Meng M, Xi J, Mo G, Chen X. 2021. Aidi injection as adjunctive treatment to gemcitabine-based chemotherapy for advanced non-small cell lung cancer: a systematic review and meta-analysis. Pharm Biol. 59(1):1260–1275. 34541998 10.1080/13880209.2021.1973038 PMC8451693

[CIT0023] Honari M, Shafabakhsh R, Reiter RJ, Mirzaei H, Asemi Z. 2019. Resveratrol is a promising agent for colorectal cancer prevention and treatment: focus on molecular mechanisms. Cancer Cell Int. 19:180. 31341423 10.1186/s12935-019-0906-y PMC6631492

[CIT0024] Hu WH, Wang HY, Kong XP, Xiong QP, Poon KK, Xu L, Duan R, Chan GK, Dong TT, Tsim KW. 2019. Polydatin suppresses VEGF-induced angiogenesis through binding with VEGF and inhibiting its receptor signaling. Faseb J. 33(1):532–544. 29989844 10.1096/fj.201800750R

[CIT0025] Hu T, Zhou C, Hu C, Sun X, Cai X, Pang Z, Su K, Cao P, Zhang X. 2016. Antioxidant activity of polygonin in ApoE^-/-^ atherosclerosis model mice. Chin Tradit Pat Med. 38:2493–2496. (Chinese) .

[CIT0026] Huang X-T, Li X, Xie M-L, Huang Z, Huang Y-X, Wu G-X, Peng Z-R, Sun Y-N, Ming Q-L, Liu Y-X, et al. 2019. Resveratrol: review on its discovery, anti-leukemia effects and pharmacokinetics. Chem Biol Interact. 306:29–38. 30954463 10.1016/j.cbi.2019.04.001

[CIT0027] Jiang KF, Zhao G, Deng GZ, Wu HC, Yin NN, Chen XY, Qiu CW, Peng XL. 2017. Polydatin ameliorates *Staphylococcus aureus*-induced mastitis in mice via inhibiting TLR2-mediated activation of the p38 MAPK/NF-kappaB pathway. Acta Pharmacol Sin. 38(2):211–222. 27890916 10.1038/aps.2016.123 PMC5309755

[CIT0028] Jin Z, Zhou B, Wang M, Wang Y. 2018. Effects of emodin in *Polygonum cuspidatum* Sieb. et Zucc on Bax and bcl-2 expression in rats with rheumatoid arthritis. Lishizhen Med Mater Med Res. 29:2572–2575. (Chinese).

[CIT0029] Jing X, Cheng W, Wang S, Li P, He L. 2016. Resveratrol induces cell cycle arrest in human gastric cancer MGC803 cells via the PTEN-regulated PI3K/AKT signaling pathway. Oncol Rep. 35(1):472–478. 26530632 10.3892/or.2015.4384

[CIT0030] Koneru M, Sahu BD, Gudem S, Kuncha M, Ravuri HG, Kumar JM, Kilari EK, Sistla R. 2017. Polydatin alleviates alcohol-induced acute liver injury in mice: relevance of matrix metalloproteinases (MMPs) and hepatic antioxidants. Phytomedicine. 27:23–32. 28314476 10.1016/j.phymed.2017.01.013

[CIT0031] Kuang S, Qi C, Liu J, Sun X, Zhang Q, Sima Z, Liu J, Li W, Yu Q. 2014. 2-Methoxystypandrone inhibits signal transducer and activator of transcription 3 and nuclear factor-kappaB signaling by inhibiting Janus kinase 2 and IkappaB kinase. Cancer Sci. 105(4):473–480. 24450414 10.1111/cas.12359 PMC4317813

[CIT0032] Lachowicz S, Oszmiański J. 2019. Profile of bioactive compounds in the morphological parts of wild *Fallopia japonica* (Houtt) and *Fallopia sachalinensis* (F. Schmidt) and their antioxidative activity. Molecules. 24(7):1436. 30979044 10.3390/molecules24071436 PMC6479739

[CIT0033] Lai Y, Zhou C, Huang P, Dong Z, Mo C, Xie L, Lin H, Zhou Z, Deng G, Liu Y, et al. 2018. Polydatin alleviated alcoholic liver injury in zebrafish larvae through ameliorating lipid metabolism and oxidative stress. J Pharmacol Sci. 138(1):46–53. 30245287 10.1016/j.jphs.2018.08.007

[CIT0034] Lee CC, Chen YT, Chiu CC, Liao WT, Liu YC, David Wang HM. 2015. *Polygonum cuspidatum* extracts as bioactive antioxidaion, anti-tyrosinase, immune stimulation and anticancer agents. J Biosci Bioeng. 119(4):464–469. 25311751 10.1016/j.jbiosc.2014.09.008

[CIT0035] Li H, Bei G, Li T, Yan G, Ten H. 2016. Experience and thinking and processing of Yao Medicine in treatment of cirrhosis ascites. Chin Arch Tradit Chin Med. 43:2580–2582. (Chinese).

[CIT0036] Li MT, Ke J, Guo SF, Wu Y, Bian YF, Shan LL, Liu QY, Huo YJ, Guo C, Liu MY, et al. 2021. The protective effect of quercetin on endothelial cells injured by hypoxia and reoxygenation. Front Pharmacol. 12:732874. 34744717 10.3389/fphar.2021.732874 PMC8564287

[CIT0037] Li X, Liu X, Gao H, Fan M, Liu K, Wang W. 2015. Study on inhibition and enzyme kinetics of different solvent extractions from *Polygonum cuspidatum* Sieb. et Zucc on xanthine oxidase. China Pharm. 26:494–496. (Chinese).

[CIT0038] Li R, Maimai T, Yao H, Liu X, He Z, Xiao C, Wang Y, Xie G. 2019. Protective effects of polydatin on LPS-induced endometritis in mice. Microb Pathog. 137:103720. 31494302 10.1016/j.micpath.2019.103720

[CIT0039] Li H, Min J, Chen Y, Li H, Zhang Y. 2020. Polydatin attenuates orbital oxidative stress in Graves’ orbitopathy through the NRF2 pathway. Chem Biol Interact. 315:108894. 31705858 10.1016/j.cbi.2019.108894

[CIT0040] Li H, Shi B, Li Y, Yin F. 2017. Polydatin inhibits cell proliferation and induces apoptosis in laryngeal cancer and HeLa cells via suppression of the PDGF/AKT signaling pathway. J Biochem Mol Toxicol. 31(7):e21900. 10.1002/jbt.21900 28266802

[CIT0041] Li Y, Yao J, Han C, Yang J, Chaudhry MT, Wang S, Liu H, Yin Y. 2016. Quercetin, inflammation and immunity. Nutrients. 8(3):167. 26999194 10.3390/nu8030167 PMC4808895

[CIT0042] Li J, Yu M, Gao Y, Zhang T, Jia H, Zhang H, Ma L, Zou Z. 2019. Anti-inflammatory active ingredient of Chinese herb *Polygoni Cuspidati Rhizoma* et Radix based on spectrum-effect relationship. Chin J Exp Tradit Med Formulae. 25:208–213. (Chinese).

[CIT0043] Li W, Zhang Q, Chen K, Sima Z, Liu J, Yu Q, Liu J. 2019. 2-Ethoxystypandrone, a novel small-molecule STAT3 signaling inhibitor from *Polygonum cuspidatum*, inhibits cell growth and induces apoptosis of HCC cells and HCC Cancer stem cells. BMC Complement Altern Med. 19(1):38. 30709346 10.1186/s12906-019-2440-9 PMC6359800

[CIT0044] Lin CJ, Lin HJ, Chen TH, Hsu YA, Liu CS, Hwang GY, Wan L. 2015. *Polygonum cuspidatum* and its active components inhibit replication of the influenza virus through toll-like receptor 9-induced interferon beta expression. PLoS One. 10(2):e0117602. 25658356 10.1371/journal.pone.0117602 PMC4319845

[CIT0045] Lin S, Wang X, Tang RW, Lee HC, Chan HH, Choi SSA, Dong TT, Leung KW, Webb SE, Miller AL, et al. 2022. The extracts of *Polygonum cuspidatum* root and rhizome block the entry of SARS-CoV-2 wild-type and omicron pseudotyped viruses via inhibition of the S-protein and 3CL protease. Molecules. 27(12):3806. 35744929 10.3390/molecules27123806 PMC9231230

[CIT0046] Liu SH. 2016. Observation on the curative effect of Huzhangshangtongding on soft tissue injury in military training. Med Front. 6:324–325. (Chinese).

[CIT0047] Liu F, Li F-s, Feng Z-m, Yang Y-n, Jiang J-s, Li L, Zhang P-c 2015. Neuroprotective naphthalene and flavan derivatives from *Polygonum cuspidatum*. Phytochemistry. 110:150–159. 25553583 10.1016/j.phytochem.2014.12.007

[CIT0048] Liu Y, Nielsen M, Staerk D, Jager AK. 2014. High-resolution bacterial growth inhibition profiling combined with HPLC-HRMS-SPE-NMR for identification of antibacterial constituents in Chinese plants used to treat snakebites. J Ethnopharmacol. 155(2):1276–1283. 25043779 10.1016/j.jep.2014.07.019

[CIT0049] Liu B, Li S, Sui X, Guo L, Liu X, Li H, Gao L, Cai S, Li Y, Wang T, et al. 2018. Root extract of *Polygonum cuspidatum* Siebold & Zucc. ameliorates DSS-induced ulcerative colitis by affecting NF-kappaB signaling pathway in a mouse model via synergistic effects of polydatin, resveratrol, and emodin. Front Pharmacol. 9:347. 29695964 10.3389/fphar.2018.00347 PMC5904535

[CIT0050] Liu D, Xie K, Yang X, Gu J, Ge L, Wang X, Wang Z. 2014. Resveratrol reverses the effects of chronic unpredictable mild stress on behavior, serum corticosterone levels and BDNF expression in rats. Behav Brain Res. 264:9–16. 24503118 10.1016/j.bbr.2014.01.039

[CIT0051] Liu D, Zhang Q, Gu J, Wang X, Xie K, Xian X, Wang J, Jiang H, Wang Z. 2014. Resveratrol prevents impaired cognition induced by chronic unpredictable mild stress in rats. Prog Neuropsychopharmacol Biol Psychiatry. 49:21–29. 24184538 10.1016/j.pnpbp.2013.10.017

[CIT0052] Liu L, Zheng G, Zhang W, Guo G, Wu M. 2014. Clinical study on treatment of carotid atherosclerosis with extraction of *Polygoni Cuspidati Rhizoma* et Radix and Crataegi Fructus: a randomized controlled trial. China J Chin Mater Med. 39:1115–1119. (Chinese). 24956862

[CIT0053] Liu TS, Zhu KJ, Yin TL, Zhu P. 2012. Multi-center clinical study of compound folium paulownieae burn oil for fresh medium and small area second degree burn. Tradit Chin Drug Res Clin Pharmacol. 23:350–353. (Chinese).

[CIT0054] Lou Y, Yu K, Wu X, Wang Z, Cui Y, Bao H, Wang J, Hu X, Ji Y, Tang G. 2021. Co-crystals of resveratrol and polydatin with l-proline: crystal structures, dissolution properties, and *in vitro* cytotoxicities. Molecules. 26(18):5722. 34577193 10.3390/molecules26185722 PMC8469398

[CIT0055] Lu H, Zhang J, Liang Y, Qiao Y, Yang C, He X, Wang W, Zhao S, Wei D, Li H, et al. 2020. Network topology and machine learning analyses reveal microstructural white matter changes underlying Chinese medicine Dengzhan Shengmai treatment on patients with vascular cognitive impairment. Pharmacol Res. 156:104773. 32244028 10.1016/j.phrs.2020.104773

[CIT0056] Luo Y, Wang C, Zhu X, Sheng G. 2016. Effect of polydatin on cell proliferation and apoptosis of leukemia cell line K562. J Zhengzhou Univ, Med Sci. 51:389–393. (Chinese).

[CIT0057] Ma T, Sheng T, Tian C, Xing M, Yan L, Xia D. 2019. Effect of ethanolic extract of *Polygonum cuspidatum* on acute gouty arthritis in mice through NLRP3/ASC/caspase-1 axis. China J Chin Mater Med. 44:546–552. (Chinese). 10.19540/j.cnki.cjcmm.20180925.001 30989921

[CIT0058] Ma C, Wang Y, Dong L, Li M, Cai W. 2015. Anti-inflammatory effect of resveratrol through the suppression of NF-kappaB and JAK/STAT signaling pathways. Acta Biochim Biophys Sin (Shanghai). 47(3):207–213. 25651848 10.1093/abbs/gmu135

[CIT0059] Ma C, Wang Y, Shen A, Cai W. 2017. Resveratrol upregulates SOCS1 production by lipopolysaccharide-stimulated RAW264.7 macrophages by inhibiting miR-155. Int J Mol Med. 39(1):231–237. 28004106 10.3892/ijmm.2016.2802

[CIT0060] Maepa M, Razwinani M, Motaung S. 2016. Effects of resveratrol on collagen type II protein in the superficial and middle zone chondrocytes of porcine articular cartilage. J Ethnopharmacol. 178:25–33. 26647105 10.1016/j.jep.2015.11.047

[CIT0061] Ming D, Songyan L, Yawen C, Na Z, Jing M, Zhaowen X, Ye L, Wa D, Jie L. 2017. Trans-polydatin protects the mouse heart against ischemia/reperfusion injury via inhibition of the renin-angiotensin system (RAS) and Rho kinase (ROCK) activity. Food Funct. 8(6):2309–2321. 28589995 10.1039/c6fo01842d

[CIT0062] Monjo AL, Pringle ES, Thornbury M, Duguay BA, Monro SMA, Hetu M, Knight D, Cameron CG, McFarland SA, McCormick C. 2018. Photodynamic inactivation of *Herpes simplex* viruses. Viruses. 10(10):532. 30274257 10.3390/v10100532 PMC6213367

[CIT0063] Montanari S, Davani L, Tumiatti V, Dimilta M, Gaddi AV, De Simone A, Andrisano V. 2021. Development of an UHPLC-diode arrays detector (DAD) method for the analysis of polydatin in human plasma. J Pharm Biomed Anal. 198:113985. 33667833 10.1016/j.jpba.2021.113985

[CIT0064] Pan B, Shi X, Ding T, Liu L. 2019. Unraveling the action mechanism of *Polygonum cuspidatum* by a network pharmacology approach. Am J Transl Res. 11(11):6790–6811. 31814888 PMC6895524

[CIT0065] Pan J, Wang H, Du X, Liu J, Zhang D. 2017. Polydatin induces human cervical cancer cell apoptosis via PI3K/AKT/mTOR signaling pathway. China J Chin Mater Med. 42:2345–2349. (Chinese). 10.19540/j.cnki.cjcmm.2017.0111 28822191

[CIT0066] Pan S, Wang Y, Wang M, Zhang Y. 2019. Effects of emodin on rheumatoid arthritis based on TNF-α-HIF-1α-iNOS-NO signaling pathway. Pharmacol Clin Chin Mater Med. 35:62–67. (Chinese).

[CIT0067] Pang N, Chen T, Deng X, Chen N, Li R, Ren M, Li Y, Luo M, Hao H, Wu J, et al. 2017. Polydatin prevents methylglyoxal-induced apoptosis through reducing oxidative stress and improving mitochondrial function in human umbilical vein endothelial cells. Oxid Med Cell Longev. 2017:7180943. 29057033 10.1155/2017/7180943 PMC5615983

[CIT0068] Park B, Lee IS, Hyun SW, Jo K, Lee TG, Kim JS, Kim CS. 2018. The protective effect of *Polygonum cuspidatum* (PCE) aqueous extract in a dry eye model. Nutrients. 10(10):1550. 30347752 10.3390/nu10101550 PMC6212923

[CIT0069] Ren L, Ou S, Chen L, Wang S, Zhao P. 2016. Anti-gouty arthritis test in rats of *Polygoni Cuspidati Rhizoma* et Radix extract and its effective parts. Chin J Exp Tradit Med Formulae. 22:111–115. (Chinese).

[CIT0070] Shen J, Xu L, Qu C, Sun H, Zhang J. 2018. Resveratrol prevents cognitive deficits induced by chronic unpredictable mild stress: sirt1/miR-134 signaling pathway regulates CREB/BDNF expression in hippocampus *in vivo* and *in vitro*. Behav Brain Res. 349:1–7. 29715537 10.1016/j.bbr.2018.04.050

[CIT0071] Sohn E, Kim J, Kim CS, Jo K, Lee YM, Kim JS. 2014. Root of *Polygonum cuspidatum* extract reduces progression of diabetes-induced mesangial cell dysfunction via inhibition of platelet-derived growth factor-BB (PDGF-BB) and interaction with its receptor in streptozotocin-induced diabetic rats. BMC Complement Altern Med. 14:477. 25495844 10.1186/1472-6882-14-477 PMC4364577

[CIT0072] Sohn E, Kim J, Kim CS, Lee YM, Kim JS. 2016. Extract of *Polygonum cuspidatum* attenuates diabetic retinopathy by inhibiting the high-mobility group box-1 (HMGB1) signaling pathway in streptozotocin-induced diabetic rats. Nutrients. 8(3):140. 26950148 10.3390/nu8030140 PMC4808869

[CIT0073] Song K, Lv T, Chen Y, Diao Y, Yao Q, Wang Y. 2018. Emodin inhibits TGF-beta2 by activating the FOXD3/miR199a axis in ovarian cancer cells *in vitro*. Oncol Rep. 39(5):2063–2070. 29512773 10.3892/or.2018.6301 PMC5928761

[CIT0074] Sougiannis AT, Enos RT, VanderVeen BN, Velazquez KT, Kelly B, McDonald S, Cotham W, Chatzistamou I, Nagarkatti M, Fan D, et al. 2021. Safety of natural anthraquinone emodin: an assessment in mice. BMC Pharmacol Toxicol. 22(1):9. 33509280 10.1186/s40360-021-00474-1 PMC7845031

[CIT0075] Su PW, Yang CH, Yang JF, Su PY, Chuang LY. 2015. Antibacterial activities and antibacterial mechanism of *Polygonum cuspidatum* extracts against nosocomial drug-resistant pathogens. Molecules. 20(6):11119–11130. 26087259 10.3390/molecules200611119 PMC6272736

[CIT0076] Sun W, Bao J, Lin W, Gao H, Zhao W, Zhang Q, Leung CH, Ma DL, Lu J, Chen X. 2016. 2-Methoxy-6-acetyl-7-methyljuglone (MAM), a natural naphthoquinone, induces NO-dependent apoptosis and necroptosis by H_2_O_2_-dependent JNK activation in cancer cells. Free Radic Biol Med. 92:61–77. 26802903 10.1016/j.freeradbiomed.2016.01.014

[CIT0077] Sun W, Qu D, Ma Y, Chen Y, Liu C, Zhou J. 2014. Enhanced stability and antibacterial efficacy of a traditional Chinese medicine-mediated silver nanoparticle delivery system. Int J Nanomedicine. 9:5491–5502. 25473286 10.2147/IJN.S71670 PMC4251751

[CIT0078] Sun D, Shen W, Wang Z, Yan T, Cheng H. 2015. Research on activity inhibition of 4 effective components in Bushen Huoxue Formula on release of inflammatory cytokines. China J Tradit Chin Med Pharm. 30:2674–2677. (Chinese).

[CIT0079] Sun B, Ye Y. 2019. The inhibitive effect of polydatin on the proliferation and invasion in lung cancer A549 cells and its mechanism. Tianjin Med J. 37:255–259. (Chinese).

[CIT0080] Sun Y, Zhao H, Bai H. 2014. *In vitro* screening of potential xanthine oxidase inhibitors by high-performance liquid chromatography. Chin J Pharm Anal. 34:1391–1396. (Chinese).

[CIT0081] Sunsong R, Du T, Etim I, Zhang Y, Liang D, Gao S. 2021. Development of a novel UPLC-MS/MS method for the simultaneously quantification of polydatin and resveratrol in plasma: application to a pharmacokinetic study in rats. J Chromatogr B Analyt Technol Biomed Life Sci. 1185:123000. 10.1016/j.jchromb.2021.123000 PMC8725175 34710805

[CIT0082] Uddin Z, Song YH, Curtis-Long MJ, Kim JY, Yuk HJ, Park KH. 2016. Potent bacterial neuraminidase inhibitors, anthraquinone glucosides from *Polygonum cuspidatum* and their inhibitory mechanism. J Ethnopharmacol. 193:283–292. 27553976 10.1016/j.jep.2016.08.026

[CIT0083] Wang J, Feng J, Xu L, Ma J, Li J, Ma R, Sun K, Wang Z, Zhang H. 2019. Ionic liquid-based salt-induced liquid-liquid extraction of polyphenols and anthraquinones in *Polygonum cuspidatu*m. J Pharm Biomed Anal. 163:95–104. 30286440 10.1016/j.jpba.2018.09.050

[CIT0084] Wang HL, Gao JP, Han YL, Xu X, Wu R, Gao Y, Cui XH. 2015. Comparative studies of polydatin and resveratrol on mutual transformation and antioxidative effect *in vivo*. Phytomedicine. 22(5):553–559. 25981921 10.1016/j.phymed.2015.03.014

[CIT0085] Wang YL, Horng CT, Hsieh MT, Chen HC, Huang YS, Yang JS, Wang GK, Chiang JH, Chen HH, Lu CC, et al. 2019. Autophagy and apoptotic machinery caused by *Polygonum cuspidatum* extract in cisplatin-resistant human oral cancer CAR cells. Oncol Rep. 41(4):2549–2557. 30720102 10.3892/or.2019.6985

[CIT0086] Wang C, Luo Y, Lu J, Wang Y, Sheng G. 2016. Polydatin induces apoptosis and inhibits growth of acute monocytic leukemia cells. J Biochem Mol Toxicol. 30(4):200–205. 26616494 10.1002/jbt.21779

[CIT0087] Wang X, Xie Y, Zhang T, Bo S, Bai X, Liu H, Li T, Liu S, Zhou Y, Cong X, et al. 2016. Resveratrol reverses chronic restraint stress-induced depression-like behaviour: involvement of BDNF level, ERK phosphorylation and expression of Bcl-2 and Bax in rats. Brain Res Bull. 125:134–143. 27346276 10.1016/j.brainresbull.2016.06.014

[CIT0088] Wang J, Yang Y, Liu Y, Zhang C, Zhang Y. 2015. Application of tincture of traditional Chinese medicine in burn treatment. China Pharm. 26:5038–5040. (Chinese).

[CIT0089] Wang C, Yang S, Lu H, You H, Ni M, Shan W, Lin T, Gao X, Chen H, Zhou Q, et al. 2015. A natural product from *Polygonum cuspidatum* Sieb. et Zucc. promotes tat-dependent HIV latency reversal through triggering P-TEFb’s release from 7SK snRNP. PLoS One. 10(11):e0142739. 26569506 10.1371/journal.pone.0142739 PMC4646521

[CIT0090] Wang Y, Yang C, Tan Y, Wang F, Xu C, Zhang M, Chen Y, Wang H. 2015. Effect of emodin from *Polygoni Cuspidati Rhizoma* et Radix on inflammation and neovascularization of RA rats. Exp Tradit Med Formulae. 21:111–115. (Chinese).

[CIT0091] Wu L, Cao K, Ni Z, Wang S, Li W, Liu X, Chen Z. 2019. Rhein reverses doxorubicin resistance in SMMC-7721 liver cancer cells by inhibiting energy metabolism and inducing mitochondrial permeability transition pore opening. Biofactors. 45(1):85–96. 30496631 10.1002/biof.1462

[CIT0092] Wu Z, Wang X, Chen M, Hu H, Cao J, Chai T, Wang H. 2019. A study on tissue-specific metabolite variations in *Polygonum cuspidatum* by high-resolution mass spectrometry-based metabolic profiling. Molecules. 24(6):1058. 30889850 10.3390/molecules24061058 PMC6471859

[CIT0093] Wu Y, Xue L, Du W, Huang B, Tang C, Liu C, Qiu H, Jiang Q. 2015. Polydatin restores endothelium-dependent relaxation in rat aorta rings impaired by high glucose: a novel insight into the PPARbeta-NO signaling pathway. PLoS One. 10(5):e0126249. 25941823 10.1371/journal.pone.0126249 PMC4420467

[CIT0094] Xia J, Yang G. 2014. Experience of TCM syndrome differentiation in the treatment of critical diseases. Chin J Basic Med Tradit Chin Med. 20:1131–1132. (Chinese).

[CIT0095] Xie Q, Yang Y, Wang Z, Chen F, Zhang A, Liu C. 2014. Resveratrol-4-*O*-d-(2′-galloyl)-glucopyranoside isolated from *Polygonum cuspidatum* exhibits anti-hepatocellular carcinoma viability by inducing apoptosis via the JNK and ERK pathway. Molecules. 19(2):1592–1602. 24473215 10.3390/molecules19021592 PMC6271899

[CIT0096] Xu Y, Hu H, Li Y, Cen R, Yao C, Ma W, Huang M, Yin Y, Gao H, Liu Y, et al. 2019. Effects of huoxin formula on the arterial functions of patients with coronary heart disease. Pharm Biol. 57(1):13–20. 31199705 10.1080/13880209.2018.1561726 PMC6586089

[CIT0097] Xu NG, Xiao ZJ, Zou T, Huang ZL. 2015. Ameliorative effects of physcion 8-*O*-beta-glucopyranoside isolated from *Polygonum cuspidatum* on learning and memory in dementia rats induced by Abeta1-40. Pharm Biol. 53(11):1632–1638. 25856718 10.3109/13880209.2014.997251

[CIT0098] Xu Y, Zhang C, Wu F, Xu X, Wang G, Lin M, Yu Y, An Y, Pan J. 2016. Piperine potentiates the effects of trans-resveratrol on stress-induced depressive-like behavior: involvement of monoaminergic system and cAMP-dependent pathway. Metab Brain Dis. 31(4):837–848. 26946512 10.1007/s11011-016-9809-y

[CIT0099] Yang F, Ding S, Liu W, Liu J, Zhang W, Zhao Q, Ma X. 2015. Antifungal activity of 40 TCMs used individually and in combination for treatment of superficial fungal infections. J Ethnopharmacol. 163:88–93. 25625353 10.1016/j.jep.2015.01.025

[CIT0100] Yang J, He Y, Li Y, Zhang X, Wong YK, Shen S, Zhong T, Zhang J, Liu Q, Wang J. 2020. Advances in the research on the targets of anti-malaria actions of artemisinin. Pharmacol Ther. 216:107697. 33035577 10.1016/j.pharmthera.2020.107697 PMC7537645

[CIT0101] Yang JW, Liu M. 2014. Research progress of traditional Chinese medicine in the treatment of drug-induced liver injury. Chinese). Chin J Tradit Med Sci Technol. 21:472.

[CIT0102] Yang L, Zhang Z, Xiang R, Gu W, Zhang H, He Y, Wang D, Liu H, Ma W, Meng F. 2019. Effect of *Polygonum cuspidatum* Sieb. et Zucc on PPARγ/NF-κB signal pathway in synovium of rats with collagen-induced arthritis. Chin J Integr Tradit West Med. 39:591–596. (Chinese).

[CIT0103] Yiu CY, Chen SY, Yang TH, Chang CJ, Yeh DB, Chen YJ, Lin TP. 2014. Inhibition of Epstein-Barr virus lytic cycle by an ethyl acetate subfraction separated from *Polygonum cuspidatum* root and its major component, emodin. Molecules. 19(1):1258–1272. 24448066 10.3390/molecules19011258 PMC6271450

[CIT0104] Zeng J, Dong X, Liu J, Guo Z, Li L. 2018. Therapeutic mechanism of polydatin on rheumatoid arthritis in rats. Nat Prod Res Dev. 30:1681–1686. (Chinese).

[CIT0105] Zeng H, Wang Y, Gu Y, Wang J, Zhang H, Gao H, Jin Q, Zhao L. 2019. Polydatin attenuates reactive oxygen species-induced airway remodeling by promoting Nrf2-mediated antioxidant signaling in asthma mouse model. Life Sci. 218:25–30. 30092299 10.1016/j.lfs.2018.08.013

[CIT0106] Zhang YK, Chen CX, Huang YC, Bian YF, Liu MY, Lv HH, Huo YJ, Han Y. 2020. Clinical efficacy of Huzhangye capsule combined with manual reduction on benign paroxysmal positional vertigo. Acad J Second Mil Med Coll. 41:1298–1301. (Chinese).

[CIT0107] Zhang L, Li Y, Gu Z, Wang Y, Shi M, Ji Y, Sun J, Xu X, Zhang L, Jiang J, et al. 2015. Resveratrol inhibits enterovirus 71 replication and pro-inflammatory cytokine secretion in rhabdosarcoma cells through blocking IKKs/NF-kappaB signaling pathway. PLoS One. 10(2):e0116879. 25692777 10.1371/journal.pone.0116879 PMC4333343

[CIT0108] Zhang Y, Wang L, Huang Q. 2015. Experimental study on topical external application of *Polygonum cuspidatum* cream in treatment of patients with tissue damage induced by calcium exteravasation. Chin Nurs Res. 29:2322–2324. (Chinese).

[CIT0109] Zhang B, Xu Y, Lv H, Pang W, Wang J, Ma H, Wang S. 2021. Intestinal pharmacokinetics of resveratrol and regulatory effects of resveratrol metabolites on gut barrier and gut microbiota. Food Chem. 357:129532. 33878586 10.1016/j.foodchem.2021.129532

[CIT0110] Zhang Q, Yuan L, Zhang Q, Gao Y, Liu G, Xiu M, Wei X, Wang Z, Liu D. 2015. Resveratrol attenuates hypoxia-induced neurotoxicity through inhibiting microglial activation. Int Immunopharmacol. 28(1):578–587. 26225925 10.1016/j.intimp.2015.07.027

[CIT0111] Zhao NW. 2016. Clinical study on Huzhangye capsules in treatment of primary hypertension with liver Yang hyperactivity. Inf Tradit Chin Med. 33:96–98. (Chinese).

[CIT0112] Zhao Y, Chen MX, Kongstad KT, Jager AK, Staerk D. 2017. Potential of *Polygonum cuspidatum* root as an antidiabetic food: dual high-resolution alpha-glucosidase and PTP1B inhibition profiling combined with HPLC-HRMS and NMR for identification of antidiabetic constituents. J Agric Food Chem. 65(22):4421–4427. 28497962 10.1021/acs.jafc.7b01353

[CIT0113] Zhao XJ, Chen L, Zhao Y, Pan Y, Yang YZ, Sun Y, Jiao RQ, Kong LD. 2022. Corrigendum to "*Polygonum cuspidatum* extract attenuates fructose-induced liver lipid accumulation through inhibiting Keap1 and activating Nrf2 antioxidant pathway" [Phytomedicine 63 (2019) 152986]. Phytomedicine. 94:153827. 34758427 10.1016/j.phymed.2021.153827

[CIT0114] Zhao Y, Jiang J, Ye J, Li Y, Li J, Yan G, Li L, Piao H. 2018. Polydatin attenuates airway inflammation in asthmatic mouse model via p38 MAPK/Nrf2/HO-1 pathway. Chin Pharmacol Bull. 34:851–856. (Chinese).

[CIT0115] Zhao J, Pan B, Zhou X, Wu C, Hao F, Zhang J, Liu L. 2022. *Polygonum cuspidatum* inhibits the growth of osteosarcoma cells via impeding AKT/ERK/EGFR signaling pathways. Bioengineered. 13(2):2992–3006. 35129428 10.1080/21655979.2021.2017679 PMC8974113

[CIT0116] Zhao XJ, Yu HW, Yang YZ, Wu WY, Chen TY, Jia KK, Kang LL, Jiao RQ, Kong LD. 2018. Polydatin prevents fructose-induced liver inflammation and lipid deposition through increasing miR-200a to regulate Keap1/Nrf2 pathway. Redox Biol. 18:124–137. 30014902 10.1016/j.redox.2018.07.002 PMC6068203

[CIT0117] Zheng L, Bao Y, Wu J, Mo J, Guo L, Wu X, Ruan L. 2017. Effects of polydatin on proliferation and differentiation of murine 3T3-L1 preadipocytes and its mechanisms. Chin Arch Tradit Chin Med. 35:2521–2525. (Chinese).

[CIT0118] Zhu C, Gu Z, Yang B, Li L, Qiu Y, Han B. 2017. Effects of polydatin-cinnamaldehyde on the gout inflammatory model induced by MSU in THP-1 cells and its mechanism. J Chin Med Mater. 40:1710–1713. (Chinese).

